# Psychoeducation for caregivers of patients with first psychotic episode

**DOI:** 10.1192/j.eurpsy.2025.559

**Published:** 2025-08-26

**Authors:** A. Magay, M. Kuzminova

**Affiliations:** 1 Mental Health Research Center of Russian Academy of Medical Sciences, Moscow, Russian Federation

## Abstract

**Introduction:**

Caregivers of patients with first psychotic episode (FPE) are under considerable stress. The onset of schizophrenia results in significant limitations for the relatives, and resulting maladaptive behavior. It is crucial to provide psychoeducation to those caring for a patient with FPE.

**Objectives:**

To assess the impact of psychoeducation on the psychological state of caregivers of patients with FPE.

**Methods:**

A total of 48 caregivers of patients with FPE (40 women and 8 men) were assessed before and after psychoeducation. Psychometric and statistical methods were used.

**Results:**

Analysis of functioning in interpersonal roles of relatives of FPE patients using the SAS-SR scale before the intervention showed significant distress in various domains (above 66 T-scores). 32.9% of relatives had impaired social interactions (withdrawal, conflicts, sensitivity to criticism). 25.4% of caregivers had strained family relationships (conflicts, guilt), and 12.4% reported difficulties in intimate relationships. 10.8% of relatives experienced problems in their relationship with the patient (overprotection combined with emotional coldness, distancing). After psychoeducation distress decreased in most areas, but some relatives still had problems of social functioning and deterioration in marital relationships. According to the SCL-90 questionnaire, distress decreased after the intervention. GSI (General Symptomatical Index) dropped from 0.69 to 0.38 (with a norm of 0.31). Anxiety and hostility also approached normal levels (from 0.68 to 0.33 and from 0.59 to 0.28, with a norm of 0.30, respectively). However, scores for paranoia (from 0.72 to 0.40, with a norm of 0.34) and depression (from 0.79 to 0.43, with a norm of 0.36) remained elevated, reflecting ongoing stress. PSDI (Positive Distress Symptomatical Index) dropped from 1.53 to 1.44. PST (Positive Symptomatical Index) dropped from 37.06 to 23.56.

After psychoeducation caregivers members’ stress coping strategies improved. Confrontation decreased (from 9 to 8 points), while social support-seeking increased (from 13 to 14 points). Avoidance behavior and distancing also decreased. Medication adherence improved: before psychoeducation 63% of caregivers had moderate adherence and 35% had low adherence. After the intervention 90% of relatives showed moderate adherence and 2% showed high adherence, and none denied the necessity of treatment.

**Conclusions:**

Psychoeducation for caregivers of patients with FPE helps them develop stress management skills, constructive communication with the patient and problem-solving strategies. The intervention reduces anxiety, stigma and improves medication adherence. The study demonstrates that psychoeducation is effective intervention that reduces the risk of relapse during the early years, contributes to the patient’s recovery.

**Disclosure of Interest:**

None Declared

